# The Interaction Effects of pri-let-7a-1 rs10739971 with *PGC* and *ERCC6* Gene Polymorphisms in Gastric Cancer and Atrophic Gastritis

**DOI:** 10.1371/journal.pone.0089203

**Published:** 2014-02-25

**Authors:** Qian Xu, Jing-wei Liu, Cai-yun He, Li-ping Sun, Yue-hua Gong, Jing-jing Jing, Cheng-zhong Xing, Yuan Yuan

**Affiliations:** 1 Tumor Etiology and Screening Department of Cancer Institute and General Surgery, the First Affiliated Hospital of China Medical University, Shenyang, Liaoning, People's Republic of China; 2 Key Laboratory of Cancer Etiology and Prevention, China Medical University, Liaoning Provincial Education Department, Shenyang, Liaoning, People's Republic of China; MOE Key Laboratory of Environment and Health, School of Public Health, Tongji Medical College, Huazhong University of Science and Technology, China

## Abstract

**Background:**

The aim of this study was to investigate the interaction effects of pri-let-7a-1 rs10739971 with *pepsinogen C* (*PGC*) and *excision repair cross complementing group 6* (*ERCC6*) gene polymorphisms and its association with the risks of gastric cancer and atrophic gastritis. We hoped to identify miRNA polymorphism or a combination of several polymorphisms that could serve as biomarkers for predicting the risk of gastric cancer and its precancerous diseases.

**Methods:**

Sequenom MassARRAY platform method was used to detect polymorphisms of pri-let-7a-1 rs10739971 G→A, *PGC* rs4711690 C→G, *PGC* rs6458238 G→A, *PGC* rs9471643 G→C, and *ERCC6* rs1917799 in 471 gastric cancer patients, 645 atrophic gastritis patients and 717 controls.

**Results:**

An interaction effect of pri-let-7a-1 rs10739971 polymorphism with *ERCC6* rs1917799 polymorphism was observed for the risk of gastric cancer (*P*_interaction_ = 0.026); and interaction effects of pri-let-7a-1 rs10739971 polymorphism with *PGC* rs6458238 polymorphism (*P*_interaction_ = 0.012) and *PGC* rs9471643 polymorphism (*P*_interaction_ = 0.039) were observed for the risk of atrophic gastritis.

**Conclusion:**

The combination of pri-let-7a-1 rs10739971 polymorphism and *ERCC6* and *PGC* polymorphisms could provide a greater prediction potential than a single polymorphism on its own. Large-scale studies and molecular mechanism research are needed to confirm our findings.

## Introduction

Individuals with similar living habits and living in similar environments possess different risks of cancer. Identifying and predicting individuals at high risk of developing cancer can indicate the need for changing living habits. Single nucleotide polymorphisms (SNPs) play a pivotal role in predicting individuals with increased cancer risk. In recent years, polymorphisms of pri-miRNA were reported to be biomarkers for predicting cancer risk, such as pri-miR-34b/c rs4938723 polymorphism [1], pri-miR-218 rs11134527 polymorphism [2] and pri-miR-938 rs2505901 polymorphism [3]. Studies have found that the number of single gene polymorphisms was associated with the risk of gastric cancer [4]. However, gastric cancer is a complex multi-step disease with many genes involved, and single SNPs have limited ability to predict gastric cancer risk [5]–[7]. Several studies have reported that gene-gene interactions are more important than the main effect of a single gene in complex diseases, such as cancers [7]–[9]. The basic research approach for gene-gene interactions is to investigate the combination of two or more polymorphisms with minor or no effects from previous single SNP studies [10], [11]. However, most previous studies have focused on the predictive role of a single gene SNP and overlooked the potential application of gene-gene interactions.

Currently, investigators mainly focus on gene SNP-SNP interactions, which can cause protein-protein interactions, and few studies have investigated interactions between miRNA polymorphisms and gene polymorphisms, which can cause protein-RNA interactions. miRNA has been reported to take part in the multi-gene network of gastric carcinogenesis [12]. The same miRNA was reported to regulate multiple target proteins, and the same proteins could be modulated by multiple miRNA [13]. As a result, the effects of miRNA and genes formed a network.

Our previous candidate gene association study investigated the association of *pepsinogen C* (*PGC*) and *excision repair cross complementing group 6* (*ERCC6*) with the risk of gastric cancer, and found that *PGC* gene rs4711690 C→G, rs6458238 G→A, and rs9471643 G→C polymorphisms had protective effects against atrophic gastritis [4]. PGC had protective effects on the normal stomach and showed low expression in gastric cancer, and the loss of protection was associated with the occurrence of gastric cancer [14]. ERCC6 is a member of the DNA repair family which participates in the repairing DNA damage in carcinogenesis [15]. *ERCC6* rs1917799 T>G polymorphism in the promoter region was associated with increased gastric cancer risk in a Chinese population [16]. Let-7a is a tumor suppressor, and recent studies found that the function of mature let-7a is closely related to the incidence and development of gastric cancer, and decreased expression of let-7a is associated with malignant biological behavior [17], [18]. However, let-7a genetic variants were not examined for their associations and gastric cancer risks. Rs10739971 is a SNP located −559 bp upstream of let-7a-1, which might be a promoter region of let-7a-1. In the Hapmap database, the minimum allele frequency of rs10739971 is ≥5% in both Europeans and Asians. But whether there is an association between rs10739971 and disease risk, and interactions between rs10739971 and other polymorphisms, remain unknown. We hypothesized that the above-mentioned variants may be optimal candidates to investigate potential SNP-SNP interactions at two or more loci contributing to gastric cancer etiology.

In this case-control study using a Chinese population, we investigated the association of pri-let-7a-1 rs10739971 with gastric cancer risk, and the interaction effects of miRNA-let-7a-1 rs10739971 polymorphism and *PGC* and *ERCC6* polymorphisms in samples of the same group, and discuss its application prospects in gastric cancer and its precancerous diseases. To our knowledge, this is the first study attempting to assess potential SNP-SNP interactions of miRNA SNPs and gene SNPs at two or more loci implicated in gastric cancer susceptibility. We hoped to find combinations of gene-gene polymorphisms that could predict the risk of gastric cancer and its precancerous diseases, and to provide experimental evidence for the early diagnosis of gastric cancer.

## Methods

### Patients

In this study, a total of 1834 individuals, which included 471 gastric cancer patients, 646 atrophic gastritis patients and 717 controls, were retrospectively recruited from patients undergoing gastroscopy examination screening in Zhuanghe region and patients who underwent gastroscopy examination or gastric surgery at the First Affiliated Hospital of China Medical University, Liaoning Province, China between 2002 and 2011. Fasting venous blood was collected and all enrolled participants were diagnosed based on their gastroscopic and histopathological examinations. Gastric cancer was diagnosed based on WHO criteria, and atrophic gastritis and superficial gastritis were classified by Sydney's classification. Eligible controls were those participants with a normal stomach or only gastritis according to gastroscopic and pathological examinations and had no other diseases.

The design of the study was approved by the Human Ethics Committee of China Medical University (Shenyang, China). Written informed consent was obtained from all participants. Medical histories (including age, sex, smoking, and alcohol consumption) were obtained by questionnaire and the records were computerized. Each individual involved in the study provided written informed consent for epidemiological investigation. Detailed participant characteristics are summarized in Table 1.

**Table 1:** The basic messages of the subjects.

Variability	CON(%)	AG(%)	GC(%)
	n = 717	n = 646	n = 471
Gender	*P*<0.001		
Male	364(50.8)	366(56.7)	320(67.9)
Female	353(49.2)	280(43.3)	151(32.1)
Age	*P*<0.001		
Mean±SD	53.11±9.83	55.06±8.94	59.04±11.15
Median	53	56	58
Range	17–85	16–82	26–87
*H.pylori-IgG*	*P*<0.001		
positive	151(21.1)	396(61.3)	245(52.0)
negative	566(78.9)	250(38.7)	226(48.0)

CON: controls; AG: atrophic gastritis; GC: gastric cancer.

### Genomic DNA extracted

Genomic DNA was extracted using a previously described method with slight modification [19]. In brief, a frozen clot (500 µL) was added to 800 µL of TE buffer (triethanolamide), mixed well and centrifuged at 10,000 × *g* for 5 min to disperse the clot. Following clot disruption, 400 µL of TE, 25 µL of 10% SDS and 5 µL of 20 mg/mL proteinase K were added and incubated at 37°C overnight. The supernatant was extracted and an equal volume of phenol was added. The tube was placed on a rotator for 15 min and then centrifuged at 10,000 × *g* for 15 min. The supernatant was removed and a second extraction was performed with the addition of an equal volume of a mixture of phenol and chloroform (1∶1). Following centrifugation, the supernatant was removed and a third extraction was performed with the addition of an equal volume of chloroform. Following centrifugation, the supernatant was absorbed and two volumes of protein precipitation solution (two volumes of absolute ethanol containing 10% 3 mol/L sodium acetate) were added and incubated for 2 h at −20°C. Each sample was centrifuged at 10,000 × *g* for 10 min. After centrifugation, the resulting DNA pellet was rinsed with 75% ethanol and centrifuged at 10,000 × *g* for 10 min. The 75% ethanol was decanted and the tube inverted on clean absorbent paper for 30 min. The resulting DNA was reconstituted in TE buffer and stored at −20°C until use.

### SNP genotyping

DNA samples were diluted to working concentrations of 50 ng/µL before genotyping. The assays, primer design, and genotyping of 29 polymorphism sites selected for a candidate gene associated study were all carried out by CapitalBio (Beijing, China) using Sequenom MassARRAY platform (Sequenom, San Diego, CA, USA) between May 2011 and December 2011 based on the manufacturer's directions[4], [20]. Five of 29 polymorphisms were further selected for interaction analysis in the present study. To evaluate the quality of the genotyping, 5% repeated samples were genotyped and the results were 100% consistent.

### Detection of H. pylori in serum

Serological tests for *H. pylori* were performed to check the status of *H. pylori* infection using ELISA (*H. pylori*-IgG ELISA kit, BIOHIT Plc, Helsinki, Finland), as described previously [21]. Positive was judged as the titer of *H. pylori*-IgG higher than 34EIU (the cut-off value given by the protocol). Briefly, serum samples were diluted 1∶200 (5 µL + 995 µL) with diluent buffer and mixed well. Then 100 µL of blank solution, calibrators, controls and diluted samples were added to wells. The plate was covered with the incubation cover and incubated for 30 min at 37°C. After incubation, the wells were washed five times with 350 µL of diluted (1∶100) washing buffer and the plate gently tapped several times on filter paper. Then 100 µL of mixed conjugate solution was added to the wells and incubated for 30 min at 37°C. After incubation, the wells were washed again and 100 µL of mixed substrate solution was added to the wells before incubating for 30 min at room temperature (20–25°C) in a dark environment. Lastly, 100 µL of mixed stop solution was added and the absorbance was read at 450 nm within 30 min.

### Statistical analysis

All statistical analyses were carried out using SPSS 16.0 software (SPSS, Chicago, IL, USA). Pearson's χ^2^ tests were used to evaluate differences between genders in case and control groups. ANOVA was performed to assess any differences between ages in the different groups. Likelihood ratio tests were performed to assess interaction effects on the risk of gastric cancer by comparing the model that only involved the main effects and the full model that also contained the interaction term. A *P*-value <0.05 for all two-sided tests was regarded as statistically significant.

## Results

### Main effect analyses of individual polymorphisms

In our previous study, we found that pri-let-7a-1 rs10739971 polymorphism was significantly associated with increased risk of atrophic gastritis (OR = 2.59, *P* = 0.018) in the smaller study population but no significant association was found in the further validation study of a larger population (unpublished data); PG*C* rs4711690, rs6458238, and rs9471643 polymorphisms were significantly associated with increased risk of atrophic gastritis (*P* = 0.093, OR = 0.75; *P* = 0.015, OR = 0.73; *P* = 0.033, OR = 0.69, respectively) [4]; *ERCC6* rs1917799 polymorphism was significantly associated with increased risk of gastric cancer (OR = 1.38, *P* = 0.035) [20].

### The two-way interaction of pri-let-7a-1 rs10739971 and PGC, ERCC6 polymorphisms in the risk of gastric cancer/atrophic gastritis

To investigate whether gene polymorphisms and miRNA polymorphism had an interaction effect, we analyzed interactions between miRNA polymorphism and each gene polymorphism. We found that miRNA polymorphism and *PGC* and *ERCC6* polymorphisms showed strong statistical association with gastric cancer or atrophic gastritis. Thus, we analyzed the interaction effect of pri-let-7a-1 rs10739971 polymorphism and *PGC* and *ERCC6* polymorphisms, and its associations, with the risks of gastric cancer and atrophic gastritis. The results indicated that pri-let-7a-1 rs10739971 polymorphism and *ERCC6* rs1917799 polymorphism had interaction effects for gastric cancer risk (*P*_interaction_ = 0.026, Table 2). This SNP pair showed increased risk of gastric cancer (OR = 2.59, 95%CI = 1.12–5.97). Pri-let-7a-1 rs10739971 polymorphism and *PGC* rs65458238 and rs9471643 polymorphisms had interaction effects for the risk of atrophic gastritis (*P*_interaction_ = 0.012 and 0.039, respectively, Table 3). For the rs65458238 polymorphism, the SNP pair showed increased risk of atrophic gastritis (OR = 2.77, 95%CI = 1.25–6.13); while the other SNP pair showed decreased risk of atrophic gastritis (OR = 0.52, 95%CI = 0.28–0.97) for rs9471643 (Table 3).

**Table 2:** The interaction of pri-let-7a-1 rs10739971 and ERCC6 rs1917799 polymorphisms in the risk of gastric cancer/atrophic gastritis^a^.

		AG vs CON(n = 646 Vs. 717)		GC vs CON(n = 471 Vs. 717)	
		let-7a-1 rs10739971		let-7a-1 rs10739971	
		GG+AG	AA	GG+AG	AA
ERCC6 rs1917799					
TT+GT	Case/Control	430/489	110/108	316/489	64/108
	OR(95%CI)	1	1.16(0.86–1.56)	1	0.92(0.65–1.29)
GG	Case/Control	88/100	18/20	69/100	22/20
	OR(95%CI)	1.01(0.73–1.37)	1.02(0.53–1.96)	1.07(0.76–1.50)	1.70(0.91–3.17)
		*P*_interaction_ = 0.941		***P*** **_interaction_ = 0.026,OR(95%CI) = 2.59(1.12**–**5.97)**	

Note: ^a^, *P* for interaction was used Logistic Regression adjusted by gender, age and *H.pylori* infection status. CON: controls; AG: atrophic gastritis; GC: gastric cancer.

**Table 3:** The interaction of pri-let-7a-1 rs10739971 and PGC polymorphisms in the risk of gastric cancer/atrophic gastritis^a^.

		AG vs CON(n = 646 Vs. 717)		GC vs CON(n = 471 Vs. 717)	
		let-7a-1 rs10739971		let-7a-1 rs10739971	
		GG+AG	AA	GG+AG	AA
PGC rs4711690					
CG+GG	Case/Control	202/247	53/55	161/247	34/55
	OR(95%CI)	1	1.18(0.77–1.79)	1	0.95(0.59–1.52)
CC	Case/Control	316/342	75/73	224/342	52/73
	OR(95%CI)	1.13(0.89–1.44)	1.26(0.87–1.82)	1.01(0.77–1.30)	1.09(0.73–1.64)
		*P*_interaction_ = 0.630		*P*_interaction_ = 0.369	
PGC rs6458238					
GA+AA	Case/Control	76/103	18/33	73/103	17/33
	OR(95%CI)	1	0.74(0.39–1.41)	1	0.73(0.38–1.40)
GG	Case/Control	442/486	110/95	312/486	69/95
	OR(95%CI)	1.23(0.89–1.70)	1.57(1.05–2.35)	0.91(0.65–1.26)	1.03(0.67–1.58)
		***P*** **_interaction_ = 0.012,OR(95%CI) = 2.77(1.25**–**6.13)**		*P*_interaction_ = 0.115	
PGC rs9471643					
GC+CC	Case/Control	222/261	66/47	166/261	38/47
	OR(95%CI)	1	1.65(1.09–2.50)	1	1.27(0.80–2.03)
GG	Case/Control	296/328	62/81	219/328	48/81
	OR(95%CI)	1.06(0.84–1.35)	0.90(0.62–1.31)	1.05(0.81–1.36)	0.93(0.62–1.40)
		***P*** **_interaction_ = 0.039,OR(95%CI) = 0.52(0.28**–**0.97)**		*P*_interaction_ = 0.196	

Note: ^a^, *P* for interaction was calculated by Logistic Regression adjusted by gender, age and *H.pylori* infection status. CON: controls; AG: atrophic gastritis; GC: gastric cancer.

### SNP-SNP interactions involving multiple SNPs using logistic regression

We further investigated the SNP-SNP interaction involving three positive SNPs (pri-let-7a-1 rs10739971–*PGC* rs6458238–*PGC* rs9471643) and the result was statistically significant (*P*_interaction_ = 0.001, Table 4). The SNP combination showed increased risk of atrophic gastritis (OR = 23.55, 95%CI = 3.73–148.71).

**Table 4:** The genotype combinations of the SNP-SNP interactions in three polymorphisms with the risk of atrophic gastritis^a^.

SNP genotypes			CON(%)	AG(%)	*P*	OR(95%CI)
pri-let-7a-1	PGC	PGC	n	n		
rs10739971	rs6458238	rs9471643	717	646		
GG+AG	GA+AA	GC+CC	28	10		1
GG+AG	GA+AA	GG	75	66	0.026	2.46(1.11–5.45)
GG+AG	GG	GC+CC	233	212	0.014	2.55(1.21–5.37)
GG+AG	GG	GG	253	230	0.014	2.55(1.21–5.36)
AA	GA+AA	GC+CC	5	9	0.016	5.04(1.36–19.68)
AA	GA+AA	GG	28	9	0.843	0.90(0.32–2.55)
AA	GG	GC+CC	42	57	0.002	3.80(1.67–8.67)
AA	GG	GG	53	53	0.013	2.80(1.24–6.33)
						***P*** **_interaction_ = 0.001, OR(95%CI) = 23.55(3.73**–**148.71)**

Note: ^a^, *P* for interaction was calculated by Logistic Regression adjusted by gender, age and *H.pylori* infection status. CON: controls; AG: atrophic gastritis.

## Discussion

Polymorphisms of genes or miRNA might serve as potential biomarkers for predicting disease risk. Previous studies have mainly focused on the association of a single gene polymorphism with disease risk, and the risk was often of weak effect (OR<1.5). As for the combination of two or more SNP-SNP interactions, the risk was often of moderate (OR≥1.5) or strong effect (OR≥2) [4], [10], [22]–[24]. The present study, for the first time, investigated the interaction effects of miRNA polymorphism and *PGC* and *ERCC6* polymorphisms in a Northern Chinese population. The study aimed to provide experimental evidence for the early diagnosis and mechanisms of gastric cancer by finding combinations of gene-gene polymorphisms that could predict the risk of gastric cancer and its precancerous diseases.

Among the SNPs from our group investigated during our candidate gene association study, *ERCC6* single-locus showed a weak effect for gastric cancer risk (OR = 1.46) [16]; the three *PGC* single-locus demonstrated a weak protective effect for atrophic gastritis risk (OR for rs4711690 = 0.75; OR for rs6458238 = 0.73; OR for rs9471643 = 0.69) [4]. We therefore hypothesized that these polymorphisms may be optimal candidates to investigate potential SNP-SNP interactions at two or more loci contributing to gastric cancer etiology. We performed the interaction effect analysis of miRNA polymorphism and gene polymorphisms and found three SNP-SNP pairs associated with diseases, of which the interaction of one pair (pri-let-7a-1 rs10739971–*ERCC6* rs1917799) was associated with gastric cancer risk, and the interactions of the other two pairs (pri-let-7a-1 rs10739971–*PGC* rs65458238, and pri-let-7a-1 rs10739971–*PGC* rs9471643) were associated with atrophic gastritis risk. *ERCC6* rs1917799 polymorphism and *PGC* rs6458238 and rs9471643 polymorphisms have been reported previously. In this study, we additionally analyzed pri-let-7a-1 rs10739971 polymorphism in the same group. The pair of pri-let-7a-1 rs10739971 polymorphism and *ERCC6* rs1917799 polymorphism had an OR of their interaction of 2.59 for gastric cancer risk, which was greater than their individual single-locus effects of 0.92 and 1.07, respectively. Similarly, the pair of pri-let-7a-1 rs10739971 polymorphism and *PGC* rs6458238 polymorphism had an OR of their interaction of 2.77 for atrophic gastritis risk, which was larger than their individual single-locus effects of 0.74 and 1.23, respectively. The pair of pri-let-7a-1 rs10739971 polymorphism and *PGC* rs9471643 polymorphism had an OR of their interaction of 0.52 for atrophic gastritis risk. Interestingly, their individual single-locus effects were contrary to the above findings, with OR of 1.65 and 1.06, respectively, which deserves further independent replication of our findings.

Because pri-let-7a-1 rs10739971 polymorphism demonstrated interaction effects with both *PGC* SNPs on atrophic gastritis risk, we further carried out interaction analysis of the three positive polymorphisms. The results showed that the OR for the risk of atrophic gastritis was 23.55 (*P* = 0.001). Generally, a single-locus has a weak effect (OR<1.5) on disease risk, and the combination of two or more SNP-SNP interactions often demonstrates a moderate (OR≥1.5) or a strong effect (OR≥2). In the present study, the OR of pri-let-7a-1 single locus was 1.61; the OR for interaction effects with *ERCC6* and *PGC* were both more than 2; the combination of the three positive loci demonstrated an even higher OR of 23.55. These outcomes indicated the application prospects of a combination of two or more SNPs as potential biomarkers to predict disease risk.

Investigating the interaction effects of miRNA polymorphisms and gene polymorphisms will contribute to the comprehensive understanding of the gene network and the role of miRNA in the pathogenic process. Findings of SNP-SNP interactions often have underlying mechanisms rather than being merely statistical results [9]. The three pairs of SNPs indicated that miRNA might interact with genes in gastric carcinogenesis. PGC protein has a protective effect in the epithelium of the normal stomach, and its expression decreased in an atrophic gastritis group when compared with a normal group [14], [25]. The exact role of PGC in atrophic gastritis is still not clear. ERCC6 is involved in the DNA repair pathway, and repeated DNA damage and repair may lead to cell carcinogenesis [15]. On the basis of the findings in the present study, it is reasonable to suggest that let-7a may participate in the processes of PGC inducing atrophic gastritis and ERCC6 inducing gastric cancer. According to results from miRNA target prediction software, there were possible binding sites of let-7a with 3′-UTR for both *PGC* and *ERCC6*. Whether the SNP-SNP interaction effects observed in this study were because of binding of miRNA and its target genes still requires further functional experiments.

This study had some limitations. First, although we had a relative large sample size of 471 gastric cancer patients, 645 atrophic gastritis patients and 717 controls, this sample size may still be inadequate for detecting interaction effects, particularly for rare alleles. Second, the mechanisms of the associated SNP-SNP interactions need to be clarified, and subsequent functional experiments are required. Third, several genome-wide association studies suggest an integration of data from genome-wide association studies of gastric cancer from Chinese populations; therefore, data should be integrated, and may be used as candidate polymorphism sites to be studied in the future. Fourth, the interaction between gene and environment for gastric cancer risk is an important factor to be considered, such as smoking and drinking. In this study, we collected some data on smoking and drinking, but there remains nearly a third of missing data from the samples. Interactions between genes and smoking and drinking behavior on gastric cancer risk should be examined.

## Conclusion

This study, for the first time, reports that pri-let-7a-1 rs10739971 polymorphism and *ERCC6* rs1917799 polymorphism might have an interaction effect on gastric cancer risk; and pri-let-7a-1 rs10739971 polymorphism might have an interaction effect with *PGC* rs6458238 and rs9471643 polymorphisms on atrophic gastritis risk. Some data have been collected providing for the possible construction of a gastric cancer network pathway. Future large-scale studies and mechanism experiments are required to confirm the findings of this study.
